# Understanding emotional and health indicators underlying the burnout risk of healthcare workers

**DOI:** 10.1371/journal.pone.0302604

**Published:** 2025-01-24

**Authors:** Elçin Güveyi, Garry Elvin, Angela Kennedy, Zeyneb Kurt, Petia Sice, Paras Patel, Antoinette Dubruel, Drummond Heckels

**Affiliations:** 1 Department of Computer Engineering, Yildiz Technical University, Istanbul, Turkey; 2 Department of Computer and Information Sciences, University of Northumbria, Newcastle Upon Tyne, United Kingdom; 3 Cumbria, Northumberland, Tyne and Wear NHS Trust, Newcastle-upon-Tyne, United Kingdom; 4 Director of Innovating For Wellbeing Limited, United Kingdom; 5 The Information School, University of Sheffield, Sheffield, United Kingdom; University of Auckland, NEW ZEALAND

## Abstract

Burnout of healthcare workers is of increasing concern as workload pressures mount. Burnout is usually conceptualised as resulting from external pressures rather than internal resilience and although is not a diagnosable condition, it is related to help seeking for its psychological sequelae. To understand how staff support services can intervene with staff heading for burnout, it is important to understand what other intrapsychic factors are related to it. A diary tool was used by staff in a region of England to self-monitor their wellbeing over time. The tool explores many areas of mental health and wellbeing and enabled regression analysis to predict which of the various factors provided the strongest indicators of burnout. Using a multiple linear regression model, burnout was found to be most associated with *depression*, *receptiveness*, *mental wellbeing*, and *connectedness* (p<0.05). It was also shown that 71% of the variance present in the response variable, i.e. burnout, explained by independent variables. Both the Spearman Rank Correlation and the Variance Inflation Factor methods found no evidence for multicollinearity in our regression models. We showed how burnout can be explained using a handful of factors including emotional and mental health indicators. The findings suggest a simple set of indicators can predict burnout and could be used for screening. The data suggests attention to four factors around social safeness, grounding and care in the self, hope and meaning, and having sufficient energy could form the basis of wellbeing programs.

## 1. Introduction

Burnout is defined as a psychological syndrome that emerges from work-related chronic stress [1]. The experience was first described as three correlated yet distinct dimensions including emotional exhaustion, depersonalisation, and therefore a reduced sense of self-actualisation [2]. The symptoms of burnout include feelings of energy depletion, negativism related to one’s job, detachment, low mood, a sense of ineffectiveness and lack of accomplishment [1]. Burnout is more likely for jobs with heavy workload, there is understaffing or the work is conflicting and unrewarding [3]. An accumulation of research has identified a high prevalence of burnout among healthcare workers [4–6]. Indeed, the health sector may be at greater risk of burnout than other professional fields as symptoms were observed in 37.9% of physicians as opposed to 27.8% in a population control sample [7]. The etiopathogenesis of burnout is multifactorial hence there are several sequential hypotheses that aim to understand the development process.

Freudenberger’s model of burnout was most recently revised into five consecutive stages that describe the developmental process [8]. The honeymoon phase specifies enthusiasm at the beginning of a job and is later followed by stagnation due to an absence of positive coping mechanisms when stressors of the job are introduced [8]. Work-life balance becomes distorted thus initiating the third stage of chronic stress characterised by feelings of failure, powerlessness, and incompetency [8]. Apathy develops with feelings of hopelessness and disparity leading to habitual burnout where one may seek help and intervention [8]. This description of burnout employs a causal sequence when coping with stress which may be preventable by enhancing control. The Job Demand-control model proposes that the risk of burnout may be implemented by an imbalance between the levels of strain and control in a work environment [9]. Low-high strain jobs can be represented through work rate, availability, time pressure, and difficulty of tasks [9]. The level of control in an employee refers to their freedom to organise and manage their workload [9]. Hence, there are several highly demanding jobs that impose stress, but they do not necessarily lead to burnout as the level of control and personal attitude of the employee are also factors.

COVID-19 magnified burnout in front-line workers as they were expected to work in stressful work environments with chronic underinvestment in the public health infrastructure, escalating workloads, inadequate support, and moral injury from being unable to provide essential care to patients. Emotional exhaustion and depersonalisation were higher in 2021 than observed in 2020, 2017, 2014 and 2011 [7]. Work-life integration declined significantly by 16.1% from 2020 to 2021 amongst physicians. Furthermore, the lack of personal protective equipment, routine testing, staff shortages, inadequate training, rapidly changing guidelines, and risk of transmission of infections to friends and family were contributory factors of burnout for healthcare workers [10–12]. This significant decline in the wellbeing of healthcare workers since the pandemic suggests that the features of a workplace are fundamental in promoting burnout.

Adjacent to workplace factors, the decision making latitude afforded of healthcare workers may also have increased the likelihood of burnout. Eder and Meyer propose the concept of self-endangering work behaviour defined as actions that aim to deal with work-related demands, yet simultaneously elicit health problems [13]. This qualitative study found that self-endangering behaviour was an essential precursor of nurses’ burnout and was based on the altruistic attitude of boosting one’s self-esteem by helping others. These behaviours may include extending work time, reducing recovery time and working overtime. The process of burnout may therefore develop through this vicious cycle and impact one’s physical and mental health. A systematic review of twelve studies found that the presence of sleep disorders was profound in nurses with a higher level of burnout [5]. Additionally, there are significant associations between burnout and depression [14]. The older the physicians and the more years they had worked, intensified the relationship between depression and burnout which may suggest that repeated and negative experiences are necessary for burnout to develop into overlapping symptoms of depression [15].

The aim of this study was to explore the correlations between various wellbeing and mental health factors and burnout, to provide a clearer understanding of the struggles of staff reporting this experience. By modelling the concept of burnout in this local population it was hoped that clearer routes to healing would emerge.

## 2. Materials and methods

### 2.1. Diary and user interface

‘My Personal Wellbeing’ is a web based self-monitoring and wellbeing screening diary facility designed for the Covid-19 pandemic and beyond. Participants were encouraged to reflect and provide descriptions of experience and their sense of wellbeing using a self-reflection diary based on a holistic integrative wellbeing model. The diary aims to help users identify and understand patterns in a number of aspects of their wellbeing to support them in managing and maintaining good wellbeing. The proactive insight gained allowed for forward planning for the NHS’ regional Staff Wellbeing Hub Services for The North East and North Cumbria region to ensure suitable and timely interventions were available to staff when they were needed the most. Data is collected using both quantitative and qualitative methods to enable objective statistical and sentiment analysis of the data.

The online diary consists of 26 wellbeing factors that are rated on a slider scale from positive to negative. Individual responses are confidential and therefore users can feel confident about being honest with their responses. In this way the diary allows self monitoring of wellbeing. Aggregated data from the diary can also be used by senior health care managers to respond to the needs of staff. Detailed information about the process of diary design was published in our previous study [16].

Table 1 indicates the questions and related codes used in the online diary that were found significant by the regression analysis (p<0.05). Items with non-significant p-values (including sleep quality, use of drugs and alcohol, moral injury, value to life, and emotional and physical wellbeing) were excluded.

**Table 1:** Questionnaire item codes and the related questions.

Item code	Question	Scale	
		Left (-10)	Right (+10)
**burnout**	Work satisfaction	Feel exhausted, unproductive, or useless at work and would avoid it if I could	Feel energised and rewarded by work
**worry**	I feel anxious and worried	A lot and it’s out of control	At peace
**apathetic**	Does the following emotion describe how you are feeling? (A checkbox is presented to users)	0 (checkbox is not clicked)	1 (checkbox is clicked)
**self compassion**	How self-compassionate are you being towards yourself?	Critical, Shaming, Frustrated	Mindful, Kind, Appreciative, Humanising
**curious**	Does the following emotion describe how you are feeling? (A checkbox is presented to users)	0 (checkbox is not clicked)	1 (checkbox is clicked)
**fear of harm from others**	Do you think you are at risk of harm from others?	I fear for my life	Safe
**avoidance**	Avoidance—Staying away from situations, people, or memories	Debilitating	Not at all
**compassion fatigue**	Ability to feel empathy or compassion	Feel burdened by the emotional suffering of others	Able to show and deliver care to others with ease
**dissociation**	Dissociation—Disconnection, numbness, emptiness, or strangely unreal sensations	Debilitating	Not at all
**perception**	Perception—Experiencing unusual things through my senses or have new concerning ideas	Debilitating	Not at all
**mental wellbeing**	Cognitive/Mental Wellbeing relating to concentration and decision making	Poor	Excellent
**depression**	I feel so low that I struggle to feel pleasure or motivation	No pleasure in anything	Interested in doing things
**receptive**	Does the following emotion describe how you are feeling? (A checkbox is presented to users)	0 (checkbox is not clicked)	1 (checkbox is clicked)
**connectedness**	Sense of connectedness with others	Lonely, unappreciated	Connected, appreciated

### 2.2. Participants and ethics declarations

The participants for this study were NHS and social care professionals, including nurses, doctors, and administrators. They were invited to complete an online diary with 26 questions about personal wellbeing. The recruitment period for this study started on the 4th January 2021 and completed on the 20th April 2022. To enter the study and gain access to the diary, participants were first required to register and confirm their consent for their data to be processed by clicking a checkbox. Participation was on a voluntary basis. Since the aim of the study was to investigate change in wellbeing over time, individuals who completed the diary only once were excluded from the analysis, leaving a total of 73 participants and 219 entries. The distribution of entries over the period of this study is shown in S1 Table. The de-identified numerical questionnaire data that does not include any personal data or text entries is provided in S2 Table.

This study has been approved by the Ethics Committee of the Faculty of Engineering and Environment at Northumbria University, Newcastle upon Tyne, with an approval reference number 23709. Informed consent was obtained from all participants or their legal guardian(s). Participants were provided details about the study and what data/information will be collected from them and were asked to agree to take part before any data was collected. All methods were conducted in accordance with relevant guidelines and regulations.

### 2.3. Regression analysis

Regression analysis is a predictive model trained to understand whether there is an association between a target variable and a set of independent variables. We can also investigate which explanatory variables are the most relevant ones to the dependent variable while predicting the relationship between them. In order to find the correlation between the dependent variable, i.e. burnout, and the independent variables, ‘regression analysis’ is used. Regression analysis can reveal the linear relationship between the target variable and the other factors. In the present study, multiple linear regression analysis was performed to explore the factors related to burnout. A two-stage regression analysis was planned: the first stage aimed to identify factors that are statistically significant for burnout (p-value under 0.05). The second stage aimed to determine the independent variables that are statistically significant in explaining the variance within the regression model, which was established using the factors identified in the first stage. All statistical analyses were conducted using R version 4.2.1 in RStudio version 2022.07.1+554. The *stats* package in R was used with the *lm* function to carry out the regression models.

The performance of the regression model was assessed using the R-squared (R^2^) metric, which indicates the proportion of variance explained by the independent variables in the model [17, 18]. A 5-Fold Cross Validation, with ten iterations, was used to validate the R-Squared values, with a dataset test / train ratio of twenty / eighty percent. In each iteration, the test dataset was fitted to the constructed model and the predicted values were obtained with *predict* function in the *stats* package. R-Squared metric was calculated for each iteration with *R2* function from caret package in R. After five iterations, average R2 values was obtained. We repeated the cross validation process 10 times to ensureconsistency in the model’s performance.

### 2.4. Analysis of covariances

ANCOVA (Analysis of Covariance) is composed of regression and ANOVA (Analysis of Variances) analyses. ANCOVA analysis helps to find out how a variable, called covariate, affects model prediction success on a continuous dependent variable with grouping based on a categorical independent variable [19]. While performing ANCOVA, one of the independent variables should be categorical, whilst the dependent variable and covariate should be continuous variables. After obtaining regression results, variables that are likely to be covariants were checked with ANCOVA. The *ancova* function from the R package *jmv* was used to perform analysis of covariances.

### 2.5. Check for multicollinearity

In multiple linear regression models, one of the important factors threatening model reliability is multicollinearity existence. Multicollinearity is defined as strong correspondence between two or more independent variables in a regression model [20]. This strong relationship can cause the regression model to providemisleading results. Correlation analysis and calculation of Variance Inflation Factor (VIF) are well-known ways to determine the presence of multicollinearity.

In this study, correlation analysis was performed using Spearman Rank Correlation, with the RStudio *stats* package’s *cor* function ("*method = spearman*"), to obtain the correlation scores, and the *gplots* package’s *heatmap*.*2* function was used to visualize the relationship between variables based on these scores. Variance Inflation Factor (VIF) [21] is another important metric for detecting dependency between variables. It is widely used in the literature to validate regression models [22, 23]. VIF score calculations were performed with the *car* package’s *vif* function in R.

## 3. Results

### 3.1. Findings from the regression model

The dataset used in the study consists of 37 questionnaire items for wellbeing and symptom-related questions. The burnout item asked diary users to rate their extent of work satisfaction by positioning a slider between two poles of ‘Feel energised and rewarded by work’ to ‘Feel exhausted, unproductive, or useless at work and would avoid it if I could’. In the multiple linear regression model, "burnout" was selected as the target variable and the other 36 items included as independent variables of the model. First, variables with a p-value below the significance level of 0.05 were selected as informative variables and then the regression model was reconstructed with only these items. Table 2 shows a summary of the burnout-targeted regression model statistical summary results, where items with p-values below 0.05 are marked as significant. From the results, the *apathetic*, *self compassion*, *compassion fatigue*, *mental wellbeing*, *depression*, *receptive*, and *connectedness items appear to be highy related* (p-value under 0.001) to *burnout*. *Thus*, *our multiple linear regression suggest these factors are important in predicting burnout (together predicting 70%* of the variation in burnout scores).

**Table 2:** Multiple linear regression model summary statistics in predicting burnout.

	*B*	*SE*	*t-value*	*p-value*
**worry**	0.1208282	0.06547753	1.8453386	0.0664306
**apathetic**	-2.4685710	0.63340665	-3.8972925	0.0001317*
**self compassion**	0.2247986	0.06579436	3.4166841	0.0007643*
**curious**	4.7569654	2.15384560	2.2085916	0.0283110*
**fear of harm from others**	-0.1480708	0.05596174	-2.6459295	0.0087786*
**avoidance**	0.1385615	0.06643903	2.0855442	0.0382577*
**compassion fatigue**	0.1943516	0.05414616	3.5893893	0.0004147*
**dissociation**	-0.1090891	0.05820600	-1.8741907	0.0623267
**perception**	0.1004212	0.05532519	1.8151084	0.0709686
**mental wellbeing**	0.2975105	0.07005322	4.2469213	0.0000329*
**depression**	0.3202704	0.06071517	5.2749640	0.0000003*
**receptive**	-7.3354672	1.68817551	-4.3452042	0.0000219*
**connectedness**	-0.2640955	0.06288644	-4.1995619	0.0000399*

*Note*: *B* stands for Beta values and *SE* represents the standart error.

* Statistically significant variables with a p-value below the 0.05.

In order to validate the regression model outcomes, an R-Squared metric was calculated on the independent test dataset with 5-Fold Cross Validation. Table 3 shows the average R2 values after ten iterations of repeated cross validation on the dataset. A mean value for R2 of 0.71 was found. This shows that more than 70% of the variance in burnout as described by extent of work satisfaction can be explained by the independent variables.

**Table 3:** Average R-Squared values for each iteration.

	Iterations									
	1	2	3	4	5	6	7	8	9	10
**Average 5-Fold**	0.703	0.709	0.722	0.712	0.710	0.703	0.720	0.718	0.695	0.711
**Average**	**0.7103 ± 0.01**									

### 3.2. ANCOVA (analysis of covariance)

Self compassion and compassion fatigue were found to be highly significant predictors in our regression model and they are known to be conceptually related [24]. We checked whether any covariates exist in our linear model using ANCOVA, and focusing on self compassion and compassion fatigue. In ANCOVA, one of the independent variables is expected to be a categorical, while the remaining predictors, including the dependent variable, should be continuous. A univariate analysis (i.e. histogram) for *self compassion* and *compassion fatigue* was performed to determine which item is more suitable for categorization. It was found that *self-compassion* was normally distributed around a zero-mean (see S1 Fig). However, this can be explained by participants leaving the sliding bar in its default position which has the value of zero. When the peak bar at the point zero is omitted, self-compassion reflects an approximate uniform distribution as it has an almost equal distribution of samples across categorical values. Thus, indicating it is suitable for discretization. Hence, *self compassion* was reshaped as a categorical variable: where values between -10 and -4 were labelled "low self compassion" (high self criticism), -3 to +3 as "medium", and 4 to 10 as "high self compassion". The ANCOVA model’s dependent variable was assigned as the diary item of *burnout using the ancova* function from the *jmv* package in R. Table 4 shows a summary of the ANCOVA model’s statistical results.

**Table 4:** Statistical summary results for ANCOVA analysis.

	*SS*	*F*	*p-value*	*PE*
**worry**	41.459154	3.854640	0.050967	1.127988
**apathetic**	156.760296	14.574696	0.000179	4.265010
**self compassion**	88.903559	4.132878	0.017399	2.418818
**curious**	46.363883	4.310655	0.039128	1.261432
**fear of harm from others**	87.439148	8.129603	0.004803	2.378975
**avoidance**	47.203180	4.388688	0.037412	1.284267
**compassion fatigue**	128.189182	11.918313	0.000676	3.487670
**dissociation**	24.910763	2.316063	0.129592	0.677752
**perception**	34.458107	3.203722	0.074954	0.937509
**mental wellbeing**	201.953081	18.776468	0.000023	5.494580
**depression**	281.446054	26.167281	0.000001	7.657362
**receptive**	181.519968	16.876712	0.000058	4.938652
**connectedness**	160.737720	14.944495	0.000149	4.373225

*Note*: *SS* stands for Sum of Squares and *PE* represents the percentage of explained variance.

The results from Table 4 show that the *apathetic*, *compassion fatigue*, *mental wellbeing*, *depression*, *receptive*, and *connectedness* variables are highly significant and have an appropriate degree of explained variance. All the variables except *worry* and *perception* have a p-value below 0.05. This means that these variables affect the prediction outcome and it’s the right choice to include them in the regression model. Thus, we concluded that the *self compassion* and *compassion fatigue* items have statistically significant p values, indicating that compassion fatigue is a covariate for the predictor categorical variable self-compassion within our linear model.

### 3.3. Correlation analysis and VIF scores

To investigate whether there is multicollinearity between the variables in the regression model, correlation analysis and Variance Inflation Factor (VIF) score calculation were performed.

**Fig 1:**
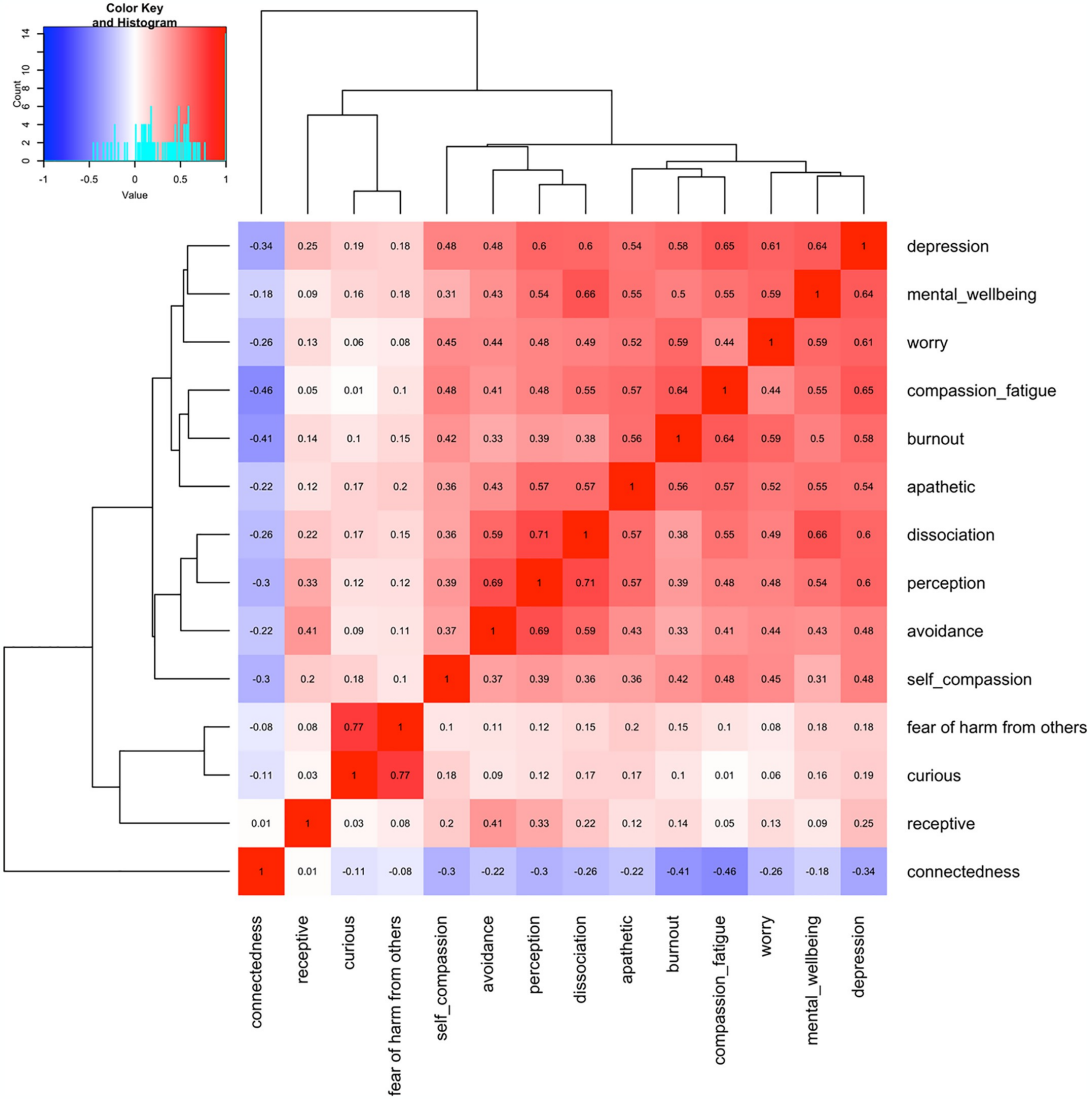
Correlation scores for variables in the regression model.

Fig 1 shows a heatmap of the results with correlation scores.

The following results that can be inferred from the heatmap:

- *curious* and *receptive* have the highest correlation in the heatmap with a coefficient of 0.77.
- *dissociation* and *avoidance* have a correlation higher than 0.7 (r = 0.71).
- *dissociation* and *perception* have almost 70% correlation (r = 0.69).
- *avoidance* and *worry* have a correlation coefficient of 0.66.
- *burnout* and *mental wellbeing* have a correlation coefficient of 0.64.
- there seem to be three distinct clusters, (i) burnout, self compassion, mental wellbeing, connectedness, worry, and depression; (ii) avoidance, dissociation, perception and compassion fatigue; (iii) fear of harm from others, receptiveness, and curiousity. While, apathetic has a weak to moderate negative correlation with the other items.

Correlation scores higher than 0.8 show that there can be multicollinearity between the variables and one of them can be kept and the rest can be removed from the model. We concluded that none of the independent variables had a correlation higher than 0.77. Thus, we hypothesised that there is no need to exclude any of the variables from the model based on the correlation scores.

Another important method of checking multicollinearity, VIF was applied to the regression model. A VIF score can start from 1, where 1 shows that there is no significant correlation between the variables of a linear model. A value between 1 and 5 indicates that the variables are moderately correlated. While, a VIF score greater than 5 means the correlation between the variables is high [25]. The VIF metric was calculated for each independent variable in the burnout-targeted regression model. Table 5 shows the results.

**Table 5:** VIF scores of the independent variables in the burnout-targeted regression model.

Item	VIF score
**worry**	2.570105
**apathetic**	1.338757
**self compassion**	2.107946
**curious**	2.569246
**fear of harm from others**	1.325771
**avoidance**	3.006439
**compassion fatigue**	1.496584
**dissociation**	2.888239
**perception**	2.130852
**mental wellbeing**	2.284351
**depression**	2.709448
**receptive**	2.581232
**connectedness**	2.173744

According to the results shown in Table 5, all VIF scores for the independent variables are between 1 and 3. This means that there is no significant relationship between the variables and that the regression model is valid. Thus, we don’t need to exclude any variable from the regression model.

## 4. Discussion

The findings of this study show that burnout, can be predicted by a small number of wellbeing factors, using simple questions. These are, how compassionate the person is being to themselves, ability to feel empathy or compassion for others, ability to concentrate and make decisions, low mood that limits pleasure, sense of connectedness to others, and the degrees of apathy, receptiveness, and curiosity. Existing models for burnout provide textural information on the concept of burnout that can be nebulous. It has been defined as a state of exhaustion characterised by a range of emotions, lack of concentration and decision making, insomnia and other physical health issues, a sense of alienation or isolation and behaviours such as using alcohol or withdrawal or lack of empathy. Our practice-based model suggests that within this particular health and care population, some of those factors in existing models, such as sleep, alcohol, irritability, physical wellbeing, were not significant. However the main factors found in this study to here aligned with the previous literature. Such wellbeing factors can have a significant impact on capacity to function and on retention of good staff [26].

Many studies in the literature on work life and patient care use the Maslach Burnout Inventory (MBI) [27] to assess burnout [28]. MBI measures burnout across three dimensions: Emotional Exhaustion, Depersonalization, and Personal Accomplishment, with each dimension including six to seven detailed questions. In contrast, our study investigates burnout using 36 different items, each highlighting a unique aspect of burnout. This approach helps us assess burnout in detail and conduct a comprehensive study. There are other studies in the literature, like ours, that use their own tools to assess burnout instead of using the MBI [29, 30]. Different from our study, Thompson’s thesis [29] focuses specifically on nurses across numerous hospitals, meanwhile our participants include NHS health and social care professionals with different occupations. The research highlights significant correlations between burnout and factors such as reduced patient care time, nurse-physician relationships, and hospital management policies. Rudman and Gustavsson [30] conducted a similar study on burnout among a more narrowed down participant group, early-career nurses, finding that those who had health issues during their final undergraduate year or depressive moods early in their careers were more prone to experience burnout.

The cluster of burnout items found in this study could form the basis of screening at work by using the small number of simple questions like those shown in Table 1. Early engagement of staff in mindful awareness of burnout as it develop, could lead to early or preventative interventions. For example, mindful Self Compassion training has been found to be helpful [31]. Opportunities to connect with others in ways that bring joy and energy may also help [32]. However burnout is known to be a consequence of working conditions. Therefore, modelling compassionate leadership and lived experience is key [33]. The UK government Health and Safety Executive has management standards covering six areas to consider to help mitigate the risk of employee burnout, including workload, choice, meaningful work, fairness, supportive teamwork, and sufficient positive recognition for staff efforts [34]. Peer support [35] and Schwartz rounds [36] have also been shown to contribute towards a healthy work culture. Additionally, resilience was also demonstrated as a moderator of the association between subjective well-being and burnout [37], as for higher levels of resilience, the negative relationship between burnout and wellbeing has been shown to have decreased among medical workers.

On the basis on the four clusters identified, S2 Fig suggests that wellbeing can be characterized by four overarching areas. One is that of social safeness—the sense of belonging and safety within a team or community. Another is the aliveness within the self: the degree to which people feeling positive about themselves and are not troubled by unusual perceptual experiences. A third area was related to cognitive aspects of hope, meaning and capacity to be focused and directed. The final cluster related to empathy for others, energy for work and capacity to be engaged. These four domains could inform the structure of future wellbeing awareness or leadership programs for employees, and suggest that attention to social factors, and cognitions as reactions to working conditions, and compassion could form the basis of cultural wellbeing initiatives.

## Supporting information

S1 Fig Distribution of the values for self_compassion. (DOCX)

S2 Fig The four overarching areas characterizing ‘wellbeing’. (DOCX)

S1 Table The number of the online diary entries in each month. (DOCX)

S2 Table Online diary questionnaire responses. (XLSX)
