# Supplementary figures and images for: Understanding emotional and health indicators underlying the burnout risk of healthcare workers
Source: PLoS One. 2025 Jan 24;20(1):e0302604. doi: 10.1371/journal.pone.0302604 (PMC11759372; doi:10.1371/journal.pone.0302604)

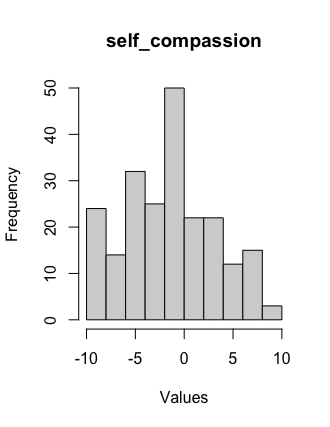


**S1 Fig. Distribution of the values of the ‘self_compassion’ item.**

Supplement: S1 Fig — (DOCX) [file pone.0302604.s001.docx]

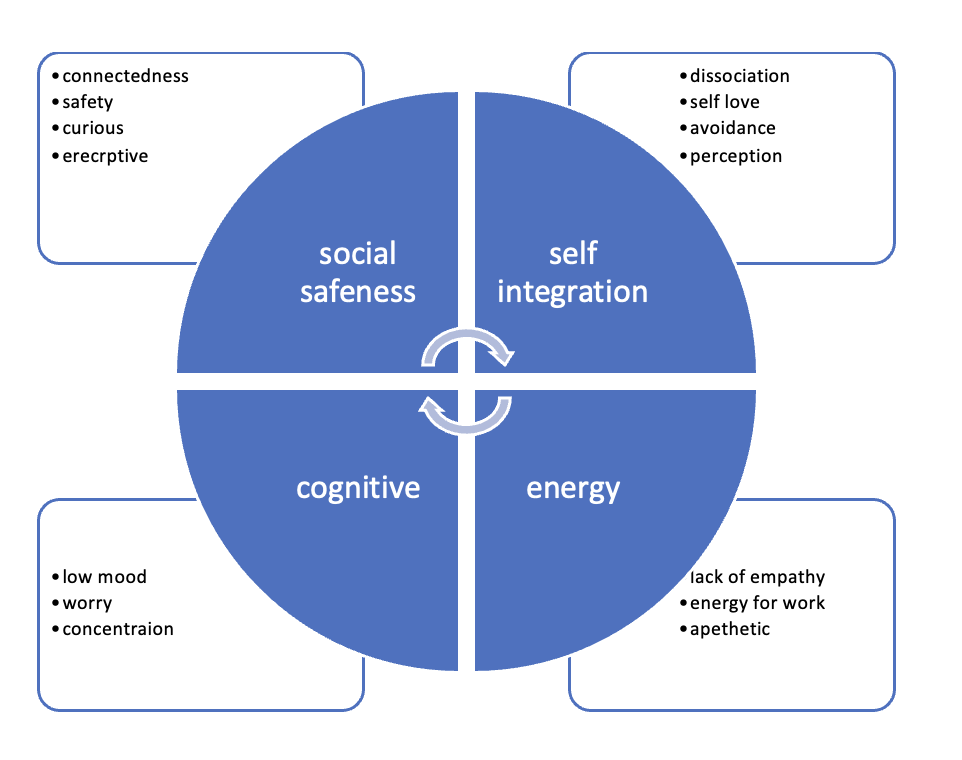


**S2 Fig. The four overarching areas characterizing ‘wellbeing’.**

Supplement: S2 Fig — (DOCX) [file pone.0302604.s002.docx]
