# Supplementary material for: Understanding emotional and health indicators underlying the burnout risk of healthcare workers
Source: PLoS One. 2025 Jan 24;20(1):e0302604. doi: 10.1371/journal.pone.0302604 (PMC11759372; doi:10.1371/journal.pone.0302604)
Supplement: S1 Table — (DOCX) [file pone.0302604.s003.docx]

**S1 Table. The number of the online diary entries in each month.**

| **Months** | **Number of Entries** |
| --- | --- |
| January 2021 | 23 |
| February 2021 | 9 |
| March 2021 | 24 |
| April 2021 | 12 |
| May 2021 | 22 |
| June 2021 | 17 |
| July 2021 | 13 |
| August 2021 | 16 |
| September 2021 | 12 |
| October 2021 | 15 |
| November 2021 | 7 |
| December 2021 | 8 |
| January 2022 | 6 |
| February 2022 | 6 |
| March 2022 | 5 |
| April 2022 | 15 |
| May 2022 | 9 |
| **Total number of survey responses** | **219** |
